# STAT1/SOCS1/3 Are Involved in the Inflammation-Regulating Effect of GAS6/AXL in Periodontal Ligament Cells Induced by *Porphyromonas gingivalis* Lipopolysaccharide *In Vitro*

**DOI:** 10.1155/2021/9577695

**Published:** 2021-10-25

**Authors:** Shengnan Zhang, Yingjun Liu, Xuekui Wang, Na An, Xiangying Ouyang

**Affiliations:** ^1^Department of Periodontology, Peking University School and Hospital of Stomatology, Beijing 100081, China; ^2^Department of General Dentistry II, Peking University School and Hospital of Stomatology, Beijing 100081, China

## Abstract

Periodontitis involves chronic inflammation of the tissues around the teeth caused by plaque and the corresponding immune response. Growth arrest-specific protein 6 (GAS6) and AXL receptor tyrosine kinase (AXL) are known to be involved in inflammatory diseases, while signal transducer and activator of transcription-1 (STAT1) and suppressor of cytokine signaling (SOCS) are related to inflammatory processes. Moreover, miRNA34a directly targets *AXL* to regulate the AXL expression. However, the specific roles of GAS6 and AXL in periodontitis remain unclear. This study was designed to explore the effect and mechanism of AXL on the expression of inflammatory cytokines induced by *Porphyromonas gingivalis* lipopolysaccharide (*P. gingivalis* LPS) in human periodontal ligament cells (hPDLCs). The effects of different concentrations of *P. gingivalis* LPS on the expression of GAS6/AXL in hPDLCs were observed. Additionally, the effect of LPS on AXL was investigated by transfection of the miRNA34a inhibitor. *AXL* was knocked down or overexpressed to observe the release of inflammatory cytokines interleukin- (IL-) 8 and IL-6. The results showed that the expression levels of GAS6 and AXL decreased after *P. gingivalis* LPS infection. Transfection of a miR-34a inhibitor to hPDLCs demonstrated a role of miR-34a in the downregulation of AXL expression induced by LPS. Moreover, *AXL* knockdown or overexpression influencing the expression of IL-8 and IL-6 was investigated under LPS stimulation. *AXL* knockdown decreased the expression of STAT1 and SOCS1/3. Overall, these results demonstrate that AXL inhibits the expression of LPS-induced inflammatory cytokines in hPDLCs and that STAT1 and SOCS1/3 are involved in the regulation of inflammation by GAS6/AXL.

## 1. Introduction

Periodontitis is a chronic infectious disease caused by dental plaque that results in the destruction of periodontal tissue. *Porphyromonas gingivalis* is prevalent in adult periodontitis patients [1], which is detected in more than 80% of all patients [2]. *P. gingivalis* lipopolysaccharide (*P. gingivalis* LPS) stimulates a proinflammatory reaction and bone resorption *in vivo* [3, 4]. *In vitro* studies have shown that multiple cells produce diverse proinflammatory cytokines, including interleukin- (IL-) 1*α*, IL-1*β*, IL-6, IL-8, and tumor necrosis factor (TNF-*α*), following *P. gingivalis* LPS challenge [5, 6].

The TAM family of receptor tyrosine kinases has three members: TYRO3, AXL receptor tyrosine kinase (AXL), and MERTK. AXL was originally isolated and identified in chronic myelogenous leukemia cells in 1991 [7–9], which is a transmembrane protein that is dependent on interaction with its ligand for activation [7]. The activation of TAM receptors depends on the binding of two ligands: growth arrest-specific 6 protein (GAS6) and protein S1 (PROS1) [10]. Among TAM receptors, GAS6 has the highest affinity for binding to AXL; however, the affinity between PROS1 and AXL has not yet been demonstrated [11, 12].

GAS6/AXL activates multiple signaling pathways in a variety of cells; plays several roles, including cell survival, proliferation, inflammation, and migration; and is involved in several pathways, including the phosphatidylinositol 3-kinase–Akt, extracellular signal–regulated kinase (ERK)1/2, and phospholipase C-*γ* pathways [13]. GAS6/AXL has also been identified as a biomarker of various human diseases, including fibrosis and inflammatory diseases [14]. GAS6 plays an important role in immune homeostasis of the oral epithelium of mice [15, 16]. *P. gingivalis* stimulates an increase in the production of type-I interferon (IFN-I) in the gingival epithelium of mice, leading to a decrease in GAS6, AXL, and PROS1 levels [17]. PROS1 was also shown to inhibit periodontal alveolar bone resorption in mice [18]. However, the role of AXL in human periodontal tissue is unclear.

Signal transducer and activator of transcription-1 (STAT1) is a member of the STAT family, which has been confirmed to play an essential role in the IFN signaling pathway in multiple studies [19]. GAS6/AXL activates STAT1 in a complex with IFNAR1-STAT1, which leads to increased expression of the suppressor of cytokine signaling-1 (SOCS1) and SOCS3 [20]. MicroRNAs (miRNAs) are short noncoding RNAs that regulate gene expression, leading to either translational repression or mRNA degradation [21]. The miR-34a was confirmed to directly target the 3′ untranslated region of *AXL* using a luciferase reporter assay [22–24]. miR-34a regulates vascular smooth muscle cell calcification by inhibiting AXL [25], and upregulation of miR-34a was shown to significantly decrease the levels of AXL in patients with rheumatoid arthritis (RA) compared with those of healthy individuals [26].

Based on this background, this study was designed to (a) evaluate the effects of miR-34a on the expression of AXL in human periodontal ligament cells (hPDLCs) stimulated by *P. gingivalis* LPS *in vitro*, (b) observe the *in vitro* effect of AXL on expression of the inflammatory cytokines IL-8 and IL-6 in hPDLCs stimulated by *P. gingivalis* LPS, and (c) investigate the role and underlying mechanisms of AXL in this process.

## 2. Materials and Methods

### 2.1. Cell Culture

hPDLCs were purchased from iCell Bioscience Inc. (Shanghai, China) and were cultured in minimum essential medium-*α* (*α*-MEM, ScienCell) supplemented with 10% fetal bovine serum (FBS), 100 IU/mL penicillin (P), and 100 *μ*g/mL streptomycin (S) at 37°C and 5% CO_2_ in a humidified incubator. The manufacturer confirmed that immunofluorescence staining of fibronectin or vimentin was positive for the cell line, and the cell purity was higher than 90%. Cells at passages 3–7 were used in subsequent experiments. “Standard” *P. gingivalis* LPS was obtained from InvivoGen (Cat. No. tlrl-pglps).

### 2.2. Stimulation Protocol

hPDLCs (2 × 10^5^ cells/well) were seeded in 6-well plates in 2 mL of *α*-MEM supplemented with 10% FBS and 1% P/S. Once cultured, hPDLCs reached 80–90% confluence; they were stimulated with varying concentrations (0 *μ*g/mL, 0.1 *μ*g/mL, 1 *μ*g/mL, and 10 *μ*g/mL) of *P. gingivalis* LPS for 24 h. The expression of GAS6/TAMs was then determined. hPDLCs at 50–70% confluence were transfected with small interfering RNAs (siRNAs), plasmids, or an miRNA inhibitor, followed by challenge with 1 *μ*g/mL *P. gingivalis* LPS for 24 h. The release of the inflammatory cytokines IL-8 and IL-6 was then observed.

### 2.3. Cell Transfection

hPDLCs (2 × 10^5^ cells/well) were seeded in 6-well plates in 2 mL of *α*-MEM supplemented with 10% FBS. When the cells reached 50–70% confluence, they were transfected with a miRNA34a inhibitor (100 nM) or miRNA negative control (mi-NC) (100 nM) using Lipofectamine 3000 (Invitrogen) according to the manufacturer's instructions. AXL siRNA (si-AXL) (20 nM) was used to knockdown *AXL* expression, and a scrambled siRNA (si-CTR) was used as a negative control, followed by challenge with 1 *μ*g/mL *P. gingivalis* LPS for 24 h. The pcDNA3.1(+) AXL plasmid (5 *μ*g) was synthesized to overexpress *AXL*. The siRNAs and plasmid were synthesized by GenePharma (Suzhou, China). The sequences of the siRNAs are listed in Table 1. The transfection efficiency was determined by measuring the gene and protein expression levels of AXL by reverse transcription-quantitative polymerase chain reaction (RT-qPCR) and western blotting assays, respectively, as described below.

### 2.4. Real-Time Quantitative PCR

Total RNA was isolated from cultured cells using a TRIzol reagent (Invitrogen) and reverse-transcribed into cDNA using the PrimeScript RT reagent kit (TaKaRa). qPCR assays of *IL-6*, *IL-8, AXL*, miRNA34a, and glyceraldehyde 3-phosphate dehydrogenase (*GADPH*) were performed using SYBR Green Master Mix (Roche). The primers used are listed in Table 2. Experiments were carried out in a 7500 Fast Real-Time Quantitative PCR system (Applied Biosystems) under the following thermocycler conditions: one cycle at 95°C for 10 min, followed by 40 cycles of 15 s at 95°C and 1 min at 60°C. The expression of target genes was normalized to that of *GAPDH* using the comparative 2^-*ΔΔ*CT^ method [27].

### 2.5. Western Blot Analysis

Cells were lysed using RIPA buffer (high efficiency) (Solarbio, China) containing 1% complete protease inhibitor and phosphatase inhibitors (Huaxingbio, China). After ultrasonication for 1 min and centrifugation for 30 min, the bicinchoninic acid assay (Thermo Fisher Scientific) was used to determine protein concentrations. Total protein (30 *μ*g) in different samples was separated by SDS-PAGE and then transferred onto polyvinylidene difluoride (PVDF) membranes (Thermo Fisher Scientific). After blocking with 5% *w*/*v* skim milk for 1 h at room temperature, the membranes were incubated with primary antibody overnight at 4°C, followed by incubation with goat-anti mouse or rabbit horseradish peroxidase-conjugated secondary antibody for 1 h at room temperature. The protein bands were imaged by chemiluminescence with a Chemidox XRS imaging system (Bio-Rad) and quantified by densitometry using ImageJ 9.0. The relative expression levels of proteins were normalized to GAPDH levels. Primary antibodies against the following proteins were used: GAS6 (Cell Signaling Technology), AXL (Bioss Antibodies), TYRO3 (Abclonal), MERTK (CUSABIO Biotech), STAT1 (Abclonal), phospho-STAT1 (Tyr701, Affinity Bioscience), suppressor of cytokine signaling (SOCS1, Affinity Bioscience), IL-6 (Abclonal), IL-8 (Abclonal), SOCS3 (Abclonal), and GAPDH (ZSGB-Bio).

### 2.6. Statistical Analysis

Each experiment was performed in triplicate with the results expressed as mean ± SD. Data analysis was performed using SPSS (version 22.0). Differences between two groups were analyzed with the unpaired *t*=test or Mann–Whitney test for normally or not normally distributed variables, respectively. Statistical analyses among multiple groups were performed using one-way analysis of variance or the Kruskal-Wallis test. Statistical significance was set at *P* < 0.05 (∗*P* < 0.05, ^∗∗^*P* < 0.01, and ^∗∗∗^*P* < 0.001).

## 3. Results

### 3.1. GAS6/AXL Expression Decreased in *P. gingivalis* LPS-Stimulated hPDLCs

**Figure 1:**
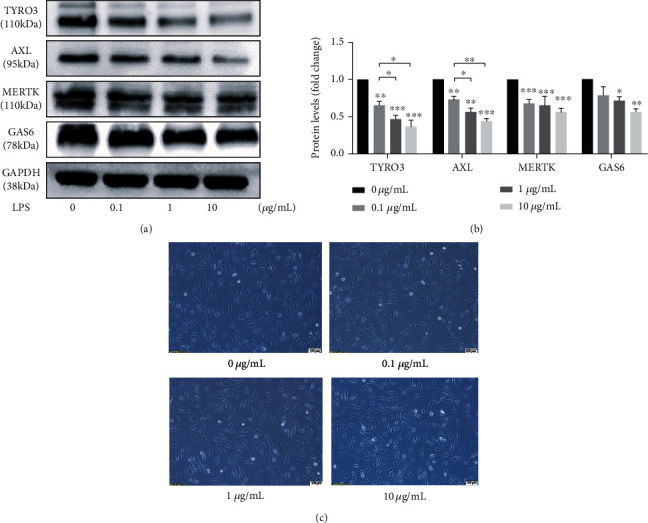
Effects of *P. gingivalis* LPS on the expression of TAM receptors and GAS6 in hPDLCs. The hPDLCs were challenged with 0, 0.1, 1, and 10 *μ*g/mL *P. gingivalis* LPS for 24 h. (a) Protein levels detected by western blotting. (b) ImageJ analysis of the western blots with a value of 1.0 assigned to the expression of each protein in the unchallenged sample. (c) Morphological images of hPDLCs treated with LPS.

GAS6, TYRO3, AXL, and MERTK were expressed in control hPDLCs without stimulation (Figure 1). However, a significant decrease in the expression level of each protein was observed following a challenge with *P. gingivalis* LPS. Furthermore, there was a concentration-dependent decrease in TYRO3 and AXL expression levels after LPS stimulation. LPS challenge at 1 *μ*g/mL for 24 h was used in subsequent experiments to mimic the periodontal inflammatory microenvironment according to previous studies [28, 29]. No morphological changes were found after incubation with *P. gingivalis* LPS for 24 h (Figure 1(c)).

### 3.2. miR-34a Inhibitor Reversed the Inhibitory Effect of *P. gingivalis* LPS on AXL Expression in hPDLCs

**Figure 2:**
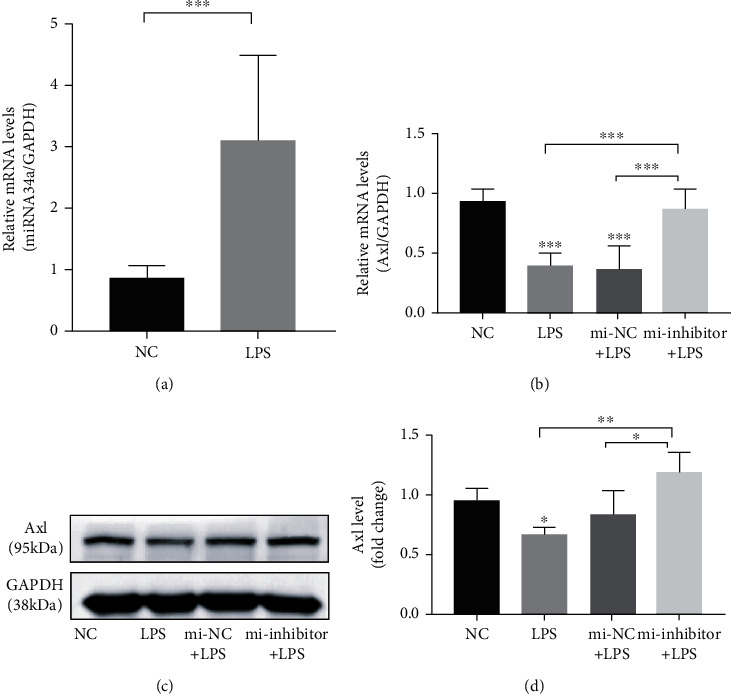
Effect of miR-34a inhibitor on the expression of AXL in hPDLCs challenged with *P. gingivalis* LPS. The level of miRNA34a after 1 *μ*g/mL *P. gingivalis* LPS stimulation (a). hPDLCs transfected with an miRNA-negative control (mi-NC) or miR-34a inhibitor were challenged with 1 *μ*g/mL *P. gingivalis* LPS. AXL expression examined by real-time PCR (b) and western blotting (c, d).

The effect of *P. gingivalis* LPS infection on AXL expression in hPDLCs was attenuated by the miR-34a inhibitor (Figure 2). As shown in Figure 2(a), the level of miRNA34a was increased after challenge of 1 *μ*g/mL *P. gingivalis* LPS stimulation. *P. gingivalis* LPS downregulated the expression of AXL at both the mRNA (Figure 2(b); *P* < 0.001) and protein (Figures 2(c) and 2(d); *P* < 0.05) levels. However, the *P. gingivalis* LPS-induced downregulation of AXL expression was reversed in hPDLCs that had been transfected with the miR-34a inhibitor (*P* < 0.05). These results suggested that miR-34a is involved in the downregulation of AXL expression induced by *P. gingivalis* LPS in hPDLCs.

### 3.3. AXL Modulated the *P. gingivalis* LPS-Induced Expression of IL-6 and IL-8 in hPDLCs

**Figure 3:**
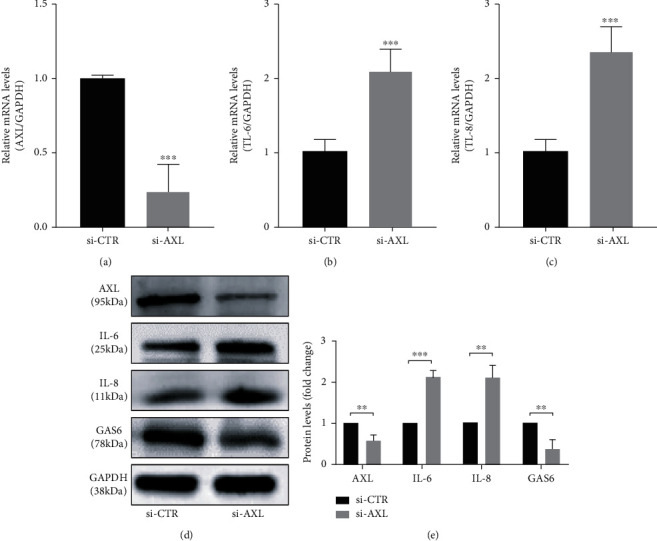
Knockdown of *AXL* enhances *P. gingivalis* LPS-induced expression of IL-6 and IL-8 in hPDLCs. (a, d) Verification of the efficiency of *AXL* knockdown at the mRNA (a) and protein (d) levels. (b–e) hPDLCs with or without *AXL* knockdown were stimulated by 1 *μ*g/mL *P. gingivalis* LPS for 24 h, and the mRNA and protein levels of IL-6 and IL-8 were examined. (d, e) Protein levels of GAS6 in hPDLCs with or without *AXL* knockdown.

*AXL* knockdown efficiencies of 76.03% and 56.97% were observed at the mRNA (Figure 3(a); *P* < 0.001) and protein level (Figures 3(d) and 3(e); *P* < 0.01), respectively, after siRNA transfection compared with the corresponding levels in the si-CTR control. hPDLCs with *AXL* knockdown exhibited increased mRNA (Figures 3(b) and 3(c)) and protein (Figures 3(d) and 3(e)) expression levels of IL-6 (*P* < 0.001) and IL-8 (*P* < 0.01). Moreover, *AXL* knockdown decreased the protein levels of GAS6 (*P* < 0.01).

**Figure 4:**
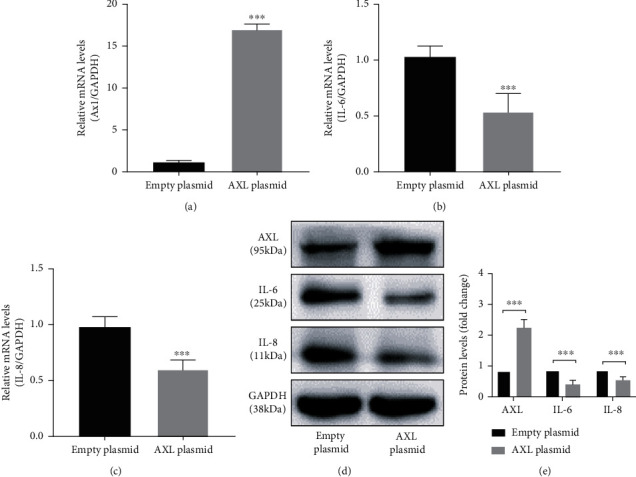
Overexpression of *AXL* reduces *P. gingivalis* LPS-induced expression of IL-6 and IL-8 in hPDLCs. (a, d) Verification of the efficiency of *AXL* overexpression at the mRNA (a) and protein (d) levels. (b–e) hPDLCs with or without *AXL* overexpression were stimulated by 1 *μ*g/mL *P. gingivalis* LPS for 24 h, and the mRNA (b, c) and protein (d, e) levels of IL-6 and IL-8 were detected.

Overexpression of *AXL* in hPDLCs resulted in approximately 17- and 2.74-fold increases at the mRNA (Figure 4(a)) and protein (Figure 4(d)) levels, respectively. The expression levels of IL-6 and IL-8 decreased in AXL-overexpressing cells compared with those transfected with the empty pcDNA3.1(+) plasmid (*P* < 0.001) (Figures 4(b)–4(e)).

### 3.4. STAT1/SOCS1/3 Are Involved in AXL-Mediated Inflammation Regulation

**Figure 5:**
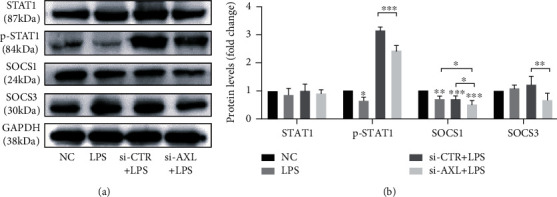
Knockdown of *AXL* affects the expression of STAT1 and SOCS1/3 in hPDLCs. The expression levels of p-STAT1 and SOCS1/3 in *AXL*-knockdown hPDLCs were detected by western blot.

hPDLCs with or without *AXL* knockdown were treated with *P. gingivalis* LPS for 24 h, and the expression levels of STAT1 and SOCS1/3 were compared with those of control cells (Figure 5). Western blotting showed that *P. gingivalis* LPS stimulation led to decreased expression of SOCS1 and increased expression of SOCS3, and the expression levels of SOCS1 and SOCS3 were both decreased significantly (*P* < 0.01) after downregulating *AXL* expression. The expression of p-STAT1 also decreased substantially after downregulating *AXL* (*P* < 0.001).

## 4. Discussion

In this study, hPDLCs challenged with *P. gingivalis* LPS exhibited reduced expression of AXL and its ligand GAS6; however, the miR-34a inhibitor reversed the inhibitory effect of *P. gingivalis* LPS on AXL expression in hPDLCs. Overexpression of *AXL* inhibited the LPS-induced expression of IL-6 and IL-8, whereas reduced *AXL* expression resulted in increased IL-6 and IL-8 levels and reduced p-STAT1, SOCS1, and SOCS3 levels.

Periodontal diseases represent multifactorial infections caused by the interaction between bacteria and host tissues or cells. *P. gingivalis* is a member of the “red complex,” which presents high detection rate at sites expressing progressing periodontitis [30]. Periodontal ligament cells produce and secrete components of the extracellular matrix such as collagen, which form the periodontal ligaments and fibers that stabilize the cementum and alveolar bone connections, enabling the periodontal ligament to regenerate after injury [31]. Periodontal ligament cells are immune cells that express IL-6, IL-8, monocyte chemoattractant protein-1 (MCP-1), and other cytokines and chemokines following inflammation [32–34]. *P. gingivalis* activates NF-*κ*B, caspase 1, and other inflammatory pathways in hPDLCs [35, 36]. We successfully established an *in vitro* periodontitis model using hPDLCs with *P. gingivalis* stimulation. Various *P. gingivalis* LPS preparations are commercially available. In contrast to “ultrapure” *P. gingivalis* LPS, “standard” *P. gingivalis* LPS shows significantly higher immunogenicity [5] and therefore may be more reflective of the complexity of local virulence factors in periodontitis.

Inflammation regulates AXL and GAS6 expression. AXL originates from hematopoietic, epithelial, and mesenchymal cells and is expressed in most human cells [7], whereas GAS6 is widely expressed in a variety of tissues such as the bone, lung, heart, and kidney, but its expression level is low in the liver [37]. *P. gingivalis* LPS inhibits the expression of AXL and GAS6 in human umbilical vein endothelial cells [38]. The inhibitory effect of *Escherichia coli* LPS on the expression of AXL in human umbilical vein endothelial cells has also been reported [39]. AXL and GAS6 expressions are upregulated in the gingival epithelium or blood vessels of mice after infection by *P. gingivalis*, whereas subsequent infections significantly reduce their expression levels [17]. However, similar levels of AXL and GAS6 have been detected in human gingival tissues with or without chronic periodontitis using qPCR [18]. This discrepancy may be explained by the multiple periodontal pathogens that contribute to chronic periodontitis. Here, we demonstrated that AXL expression in hPDLCs decreased in a concentration-dependent manner after challenge with *P. gingivalis* LPS. Both 1 *μ*g/mL and 10 *μ*g/mL *P. gingivalis* LPS inhibited the production of GAS6 and TAMs in hPDLCs. As previously reported, hPDLCs treated with *P. gingivalis* LPS (5 *μ*g/mL) showed a significant reduction of viable cells with respect to the control group [35]. Referring to numerous studies involving *P. gingivalis* LPS [28, 29, 40], we used a challenge concentration of 1 *μ*g/mL for 24 h in subsequent experiments to best mimic the periodontal inflammatory microenvironment.

Studies of AXL expression in tumor and autoimmune diseases revealed that AXL is regulated by a variety of miRNAs, including miR-34a, miR-199a, and miR-92b [25, 41–43]. The level of miR-34a in dendritic cells derived from patients with RA is higher than that in healthy individuals [26]. In this study, transfection of hPDLCs with miR-34a reversed the reduction of AXL expression in hPDLCs induced by *P. gingivalis* LPS. The AXL receptor exerts anti-inflammatory effects in a variety of cells and tissues. *Axl^−/−^* mice displayed accelerated progression of Japanese encephalitis, increased nerve cell death, expanded inflammatory cell infiltration, and increased proinflammatory cytokine levels compared with those of control mice [44]. Anti-AXL antibody therapy reduces the severity of experimental autoimmune encephalomyelitis in mice [45]. Moreover, AXL mediates LPS-induced endothelial inflammatory activation and *AXL* knockdown increases the expression of intercellular adhesion molecule 1 (ICAM-1) and the inflammatory factors IL-6 and IL-8 [39]. Supplementation of the AXL ligand GAS6 as a therapeutic drug inhibited inflammatory diseases of the central nervous system by inhibiting the upregulation of TNF-*α* in *Axl^−/−^* microglia induced by *E. coli* LPS [46] and by increasing the expression of IL-10 and transforming growth factor-beta (TGF-*β*) [47]. However, a proinflammatory effect of GAS6/AXL was observed in the liver and kidney [48]. In this study, the expression of IL-6 and IL-8 in hPDLCs increased and decreased after *AXL* knockdown and overexpression, respectively. GAS6 deficiency has been reported to reduce the expression of TNF-*α* and MCP-1 and was suggested to prevent liver inflammation, steatohepatitis, and fibrosis in mice [49]. The different effects of GAS6/AXL on inflammation observed in these studies may be related to its tissue-specific effects.

Inhibition of Toll-like receptor- (TLR-) mediated inflammation exerted by GAS6/AXL has also been demonstrated [50]. SOCS plays an important role in the negative regulation of TLR- and cytokine receptor-mediated inflammatory pathways. GAS6/TAM is related to the inhibition of TLR-mediated inflammatory pathways through SOCS1 and SOCS3 induction [50, 51]. GAS6 activates STAT1 via TAM receptors; in complex with IFNAR1-STAT1, STAT1 is modified (likely phosphorylated) and translocate to the nucleus, leading to the induction of SOCS1 and SOCS3 to regulate the inflammatory response [50]. Our results are consistent with these previous findings, showing that the expression of SOCS1/SOCS3 and p-STAT1 substantially decreased after downregulation of AXL expression in hPDLCs challenged with *P. gingivalis* LPS. This is considered to be the effect of the transfection agent Lipofectamine 3000, which may explain the increase of p-STAT1 after transfection with the control siRNA; however, the increase of p-STAT1 was decreased after transfection with the si-AXL by Lipofectamine 3000. In a mouse model of periodontitis induced by *P. gingivalis*, the loss of MyD88 in TLR signaling significantly reduced the expression of GAS6, AXL, and PROS1 [17]. PROS inhibits the production of TNF-*α*, IL-6, IL-1*β*, MMP9/2, and RANKL induced by *P. gingivalis* LPS through SOCS1/3 and STAT1/3 in human gingival epithelial cells *in vitro* [18]. Damage to the GAS6/AXL signaling pathway results in decreased expression of SOCS1/3, which ultimately causes uninhibited expression of IFN-I in epithelial cells. Therefore, further experiments are needed to better understand the role of STAT1 in inflammation regulation in this context, such as by examining the effect of specific STAT1 inhibitors on the changes of IL-6 and IL-8. Moreover, *in vivo* studies are also planned to confirm these effects. Notably, AXL has been shown to suppress macrophage inflammatory function by inducing Twist proteins that suppress NF-*κ*B–dependent TNF-*α* gene activation [19]. Therefore, the Twist/NF-*κ*B pathway will be the focus of our future research on the role of AXL in the inflammatory regulation in periodontitis.

## 5. Conclusions

In summary, AXL expression in hPDLCs induced by *P. gingivalis* LPS regulated the expression of the inflammatory cytokines IL-6 and IL-8 via the STAT1/SOCS pathway. miR-34a reversed the inhibition of AXL expression by *P. gingivalis* LPS challenge in hPDLCs. This *in vitro* study thus deepens our understanding of the molecular mechanism involved in *P. gingivalis* LPS-induced inflammation in hPDLCs, which requires further confirmation *in vivo*.

## Figures and Tables

**Table 1:** siRNA sequences used in this study.

siRNA	Sense strand (5′–3′)	Antisense strand (5′–3′)
si-AXL	GGGUGGAGGUUAUCCUGAATT	UUCAGGAUAACCUCCACCCTT
si-CTR	UUCUCCGAACGUGUCACGUTT	ACGUGACACGUUCGGAGAATT

**Table 2:** Sequences of primers used for qPCR.

Genes	Primer sequences (5′–3′)
*AXL*	Forward: ATCAGCTTCGGCTAGGCAG
	Reverse: TCCGCGTAGCACTAATGTTCT
*GAPDH*	Forward: GGGGAGCGAGATCCCTCCAAAATCAAGTGGGG
	Reverse: GGGTCATGAGTCCTTCCACGATACCAAAGTTG
*IL-6*	Forward: GATTCAATGAGGAGACTTGCC
	Reverse: TGTTCTGGAGGTACTCTAGGT
*IL-8*	Forward: ACTGAGAGTGATTGAGAGTGGAC
	Reverse: AACCCTCTGCACCCAGTTTTC
miRNA34a	Forward: TGAGTGTTTCTTTGGCAGTG
	Reverse: CTGATTGCTTCCTTACTATTAG

## Data Availability

All datasets generated for this study are included in the article.
